# The role of RAD51 regulators and variants in primary ovarian insufficiency, endometriosis, and polycystic ovary syndrome

**DOI:** 10.1093/narmme/ugae010

**Published:** 2024-09-28

**Authors:** Maggie Witham, Sarah R Hengel

**Affiliations:** Department of Biology, Tufts University, Medford, MA 02155, USA; Department of Biology, Tufts University, Medford, MA 02155, USA

## Abstract

The study of RAD51 regulators in female reproductive diseases has novel biomarker potential and implications for therapeutic advancement. Regulators of RAD51 play important roles in maintaining genome integrity and variations in these genes have been identified in female reproductive diseases including primary ovarian insufficiency (POI), endometriosis, and polycystic ovary syndrome (PCOS). RAD51 modulators change RAD51 activity in homologous recombination, replication stress, and template switching pathways. However, molecular implications of these proteins in primary ovarian insufficiency, endometriosis, and polycystic ovary syndrome have been understudied. For each reproductive disease, we provide its definition, current diagnostic and therapeutic treatment strategies, and associated genetic variations. Variants were discovered in *RAD51*, and regulators including *DMC1, RAD51B*, *SWS1*, *SPIDR*, *XRCC2* and *BRCA2* linked with POI. Endometriosis is associated with variants in *XRCC3*, *BRCA1* and *CSB* genes. Variants in *BRCA1* were associated with PCOS. Our analysis identified novel biomarkers for POI (*DMC1* and *RAD51B*) and PCOS (*BRCA1*). Further biochemical and cellular analyses of RAD51 regulator functions in reproductive disorders will advance our understanding of the pathogenesis of these diseases.

## Graphical Abstract

**Graphical Abstract:**
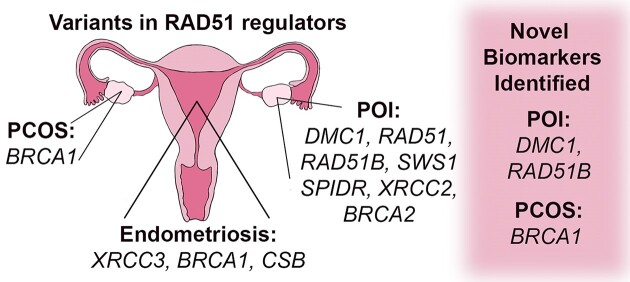

## Introduction

Understanding the direct link between female reproductive diseases and the molecular mechanisms that define them is a promising new area of study with potential for new therapeutics and interventions. Specifically, we focused on primary ovarian insufficiency (POI), endometriosis, and polycystic ovary syndrome (PCOS) in our review. RAD51 is an essential protein that functions during homology search and strand exchange to mediate templated, high-fidelity repair of the human genome (1). Moreover, mutations in regulators/modulators of human RAD51 have been identified to play important roles in hereditary breast and ovarian cancers (2,3).

The RAD51 protein is modulated by many proteins. In this review we focused on regulators of RAD51 which stimulate RAD51 functions including but not limited to ssDNA binding, ATPase activity, homology search and strand exchange activities, and replication stress resolution (3,4). During RAD51-templated strand exchange activities, the physiological ssDNA substrate is coated by a protein called replication protein A (RPA) (5). RAD51 modulators which enhance RPA displacement from the ssDNA templates and stimulate RAD51 loading on ssDNA are called recombination mediators (6,7). Recombination mediators are distinct from other RAD51 regulators as few proteins can enhance RPA displacement from the ssDNA template. To date, recombination mediators include BRCA2, PDS5B and the RAD51 paralogs (1,7–9). The RAD51 paralogs are important in maintaining genome integrity by stimulating RAD51 functions during double strand break (DSB) repair, replication stress responses, and template switching (TS) mechanisms utilized in highly dividing cells (10–16).

The RAD51 paralogs include DNA repair proteins RAD51B, RAD51C, RAD51D, XRCC2, XRCC3 and SWSAP1; all of which stimulate RAD51 functions through divergent mechanisms (10,11,17). The RAD51 paralogs share Walker A and B motifs that enable ATPase activity to regulate RAD51 protein conformation and function (4,16). Together, the RAD51 paralogs form complexes BCDX2, CX3 and the SWSAP1–SWS1 complex (16). Both BRCA1 and BRCA22 as well as proteins comprising the BCDX2 and CX3 complexes are present on breast cancer screening panels, but their roles in reproductive diseases remain less mechanistically defined (3,18). Variations in the RAD51 regulators occur as genetic mutants or by an increase or decrease in copy number that can result in protein amplification or depletion, respectively (16,19).

Importantly, we found that mutations in the RAD51 regulators can result in infertility. Given that these proteins are also commonly mutated in reproductive cancers, it is likely they have a role in maintaining a functional reproductive system (16,19–21). Understanding the intersection of mutations found in the RAD51 regulators in POI, endometriosis and PCOS will likely illuminate mechanisms that affect molecular medicine.

Biomarkers are defined from observations of disease-associated molecular changes that may be useful in disease diagnosis (22). We have further defined a biomarker to meet these three criteria: the gene variant is identified in a patient population, the variant protein is biochemically characterized, and cellular functional analysis has been performed. By the above criteria, we have identified three novel biomarkers for diagnosis including *DMC1* and *RAD51B* for POI as well as *BRCA1* for PCOS. Additionally, there are solved structures to further support claims for these genes as novel biomarkers. We also identified strong genetic biomarkers for POI to include *SWS1*, *SPIDR*, and *BRCA2* as they have not met the above biomarker criteria, but these genes have been mutated in multiple ethnically diverse populations.

We would like to highlight that the terms ‘female’, ‘woman’ and ‘women’ are used throughout this manuscript, but these reproductive disorders can affect anyone with a uterus regardless of gender identity.

**Primary ovarian insufficiency**: Primary ovarian insufficiency (POI) is a disease that causes premature ovarian failure in women under the age of 40, which can lead to the development of early menopause that manifests primarily as amenorrhea and infertility (23–25). A comprehensive population study conducted between 1995 and 1997 across various locations in the United States identified the overall prevalence of POI to be 1.1% (26). It is important to note that African American, Black, and Hispanic women had higher rates of POI compared to Caucasian women (26). The cause of this difference in POI prevalence is currently undefined in the literature.

Some possible etiologies of POI include altered hormone levels, ovarian follicle dysfunction/exhaustion, meiotic defects resulting from mutated DMC1 or RAD51 regulators, and environmental factors (27). On the molecular level, POI is indicated by high levels of follicle stimulating hormone (FSH) and low estradiol (E2). The elevated levels of these hormones cause symptoms such as irregular menses, infertility, delayed puberty, smaller ovaries, a lack of primary follicles, and urogenital problems like vaginal dryness or itching, as well as secondary conditions such as cardiovascular disease, osteoporosis, and type II diabetes (23,24,28). POI patients often experience premature ovarian failure due to either ovarian follicle dysfunction, a failure of maturation of ovarian follicles, or follicle depletion, a lack of follicles in the ovary (24,28). A high familial disease presence among POI patient cohorts in addition to a strong association between POI and other genetic diseases suggests that POI may cause by heritable genetic factors (23,24).

Oogenesis is the process of oocyte maturation and is dysregulated in women with POI. Women are born with a fixed number of oocytes which is dependent on proper germ cell division and meiosis (29). Beginning in embryogenesis, some germ cells enter meiosis and arrest in the diplotene stage of meiosis I until maturation (24,29). During meiotic prophase I, the cell generates intentional DSBs which are ultimately repaired through homologous recombination (HR). HR is a DSB repair mechanism that utilizes a template for accurate repair. In meiosis, homologous chromosomes in the diploid cell align and form synaptonemal complexes that contain DSBs (30). Then, RAD51 filaments search for homologous sequences that can be resolved via a crossover event to exchange genetic material (30–32). Crossovers occur at least once in each pair of homologous chromosomes during meiosis (33,34). These crossover events are integral to cellular diversity and survival (29,31). Therefore, functional RAD51 (in meiotic and mitotic cells) and DMC1 (in meiotic cells) are essential for HR and effective chromosome segregation during germ cell development (28,32,35,36). A comprehensive analysis of 8 essential regulators of prophase I and meiotic entry which have variants associated with female infertility are thoroughly described in Biswas *et al.* (37). These chromosomal abnormalities in germ cells such as breaks or deletions cause DNA damage and having defective DNA repair pathways can decrease the functional follicle pool and increase the risk of POI (38–40). Moreover, if there are mutations or copy number variations in RAD51 regulators, then DSBs result in chromosome mis-segregation during meiosis. This can lead to inadequate oocyte development or aneuploidy which can ultimately result in POI (29,41). Overall, the exact cause of the disease is unclear but recent publications have shed light on a connection to DNA replication and repair (23,24,42–44).

Current Diagnostic and Treatment Practices for POI: Diagnostic criteria range across the literature and in clinical practice, however, hormone levels and menstrual cycle abnormalities are commonly used to diagnose patients with POI (23). The diagnostic criteria for POI by the American College of Obstetrics and Gynecologists (ACOG) state that a patient must have menstrual irregularities for at least 3 consecutive months as well as abnormal FSH and E2 levels in tests at least 1 month apart (45,46). The European Society of Human Reproduction and Embryology (ESHRE) defines POI as oligo- or amenorrhea present for at least four months and two instances of elevated levels of FSH at least one month apart (23,47). Most diagnostic criteria include a combination of these two components of menses disruption and altered levels of reproductive hormones. It is important that a patient with POI is diagnosed early due to the various health complications of this disease that compound over time, especially related to cardiovascular, bone and sexual health (45).

Treatment strategies for POI mainly focus on the administration of hormone replacement therapies to offset the dysregulation of reproductive hormones in patients with this disease. Treatments depend on time of diagnosis and whether a patient has completed puberty (45). In either situation, hormone replacement therapies are required for the patient's lifetime to manage this disease until the age of menopause. Pre-pubertal patients are initially given E2 and are slowly introduced to progesterone once breast development is complete (45). After puberty, patients are given a low dose of daily estradiol as well as cyclic progesterone treatments (45,48). Often, POI patients will not use oral contraceptives (OCs) to manage symptoms because they contain higher E2 concentrations than necessary and OCs have undesirable side effects (45,48). Managing infertility is another concern for POI patients. Currently, there are limited techniques used to accurately predict follicle count and fertility of patient ovarian reserves. They include measuring serum E2 or anti-Müllerian hormone (AMH) levels or with transvaginal ultrasounds, but all are variable and may not be completely predictive of a patient's fertility (27,45,49,50). Identification of a molecular marker for less invasive assessments may be beneficial for these patients.

DMC1, RAD51, RAD51 regulators, and RAD51 mediators in POI: Of the three reproductive diseases focused on in this review, POI has the largest data set including seven missense variants (codon change resulting in variant amino acid), three frameshift mutations (deletion of one nucleotide), four premature stop codons and one aberrant splicing resulting in a missense variant. Focusing on each protein specificially: DMC1 (3 missense variants), RAD51 (1 missense variant), RAD51B (1 frameshift deletion), SWS1 (2 missense variants and 1 premature termination), SPIDR (3 premature termination), XRCC2 (1 aberrant splicing), and BRCA2 (1 missense variant and 2 frameshift deletions) as described in (Table 1). For each gene listed below, we describe the families identified with these variants in addition to any characterization of their variant and its functional implications.

**Table 1.** Mutations in DMC1, RAD51, RAD51 regulators, and RAD51 mediators in primary ovarian insufficiency

Gene symbol	Protein	Chromosomal mutation	Protein mutation	Mutation effect	Reference
*DMC1*	DMC1 (disrupted meiotic cDNA 1)	c.106G > A	D36N	missense	He *et al.* (54)
		c.28delG	E10N	missense	Cao *et al.* (56)
		c.33551A > G	M200V	missense	Mandon-Pepin *et al.* (52)
*RAD51*	RAD51 (Radiation Isolate 51)	c.203A > 3G	E68G	missense	Luo *et al.* (25)
*RAD51B*	RAD51B (Radiation Isolate 51 B)	c.92delT	N/A	frameshift mutation (deletion)	Franca *et al.* (31)
*ZSWIM7 (SWS1)*	SWS1 (SWIM domain-containing protein 7)	c.173C > G	S58*	premature termination	McGlacken-Byrne *et al.* (30)
		c.38T > C	L13P	missense	Yatsenko *et al.* (66)
		c.176C > T	S59L	missense	Hussain *et al.* (65)
*SPIDR (KIAA0146)*	SPIDR (Scaffolding protein involved in DNA repair)	c.839G > A	W280*	premature termination	Smirin-Yosef *et al.* (28)
		c.814C > T	R272*	premature termination	Heddar *et al.* (68)
		c.2002C > T	Q668*	premature termination	Rouen *et al.* (70)
*XRCC2*	XRCC2 (X-ray repair cross complementing 2)	c.41T > C	L14P	aberrant splicing	Zhang *et al.* (72)
*BRCA2*	BRCA2 (Breast cancer type 2 susceptibility protein)	c.7579delG	V2527X	frameshift mutation (deletion)	Weinberg-Shukron *et al.* (75)
		c.9693delA	S323fs16	frameshift mutation (deletion)	Weinberg-Shukron *et al.* (75)
		c.8524C > T	R2842C	missense	Caburet *et al.* (76)

This table contains information about variants associated with POI, focusing on gene and protein name, chromosomal location, protein mutation and the references for each study.

**Figure 1.**
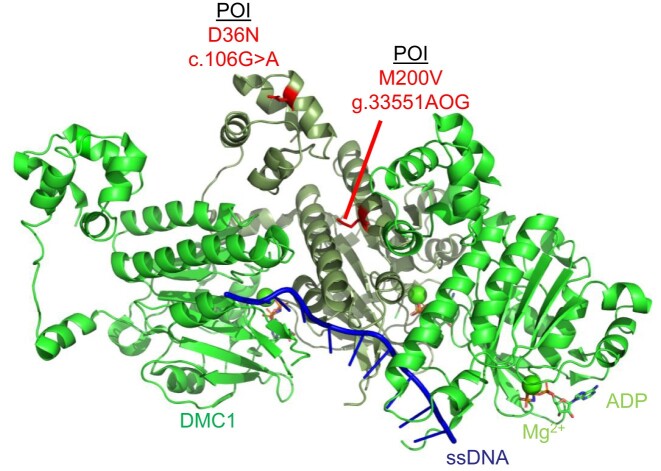
Schematic of the DMC1 (PDB 7C9C) pre-synaptic complex on ssDNA which shows the DMC1 protein protomer on ssDNA (green and sage). The DMC1 variant c.106G > A variant (D36N) and variant g.33551AOG (M200V) are highlighted in red. ssDNA is depicted in blue, magnesium ions are shown as spheres in green, and ATP molecules are shown as sticks according to ion color with a green backbone.

**DMC1:** Disrupted meiotic cDNA (DMC1) is the meiosis-specific recombinase which is required the meiosis-specific chromosomal structure (SC) and for progression beyond meiotic prophase I (36,51). Mutational variants in *DMC1* were first identified by Mandon-Pepin *et al.* at the Pitié-Salpetriere Hospital in Paris, France in 44 women with premature ovarian failure who had primary or secondary amenorrhea with a DMC1 protein M200V mutation (52). These patients had high FSH, low estradiol, a small uterus, and ovaries with no follicles. Then, in 2008, the same group identified another homozygous patient with the *DMC1* variant g.33551AOG (M200V) from Senegal in the Sarakholé group (52). This African woman from presented with high FSH, low estradiol, and upon ultrasound displayed small uterus, small ovaries, and an absence of ovarian follicles. After further study of 32 additional patient samples of Senegal origin and from the same Sarakolé ethnic group of the initial patient at the Pasteur Institute in Dakar, researchers additionally found two other patients with a heterozygous substitution at the same genome position in the *DMC1* gene (52). After *in silico* evaluation using the PolyPhen program, Mandon-Pepin *et al.* initially predicted that this *DMC1* variant is likely pathogenic. Importantly, a thorough biochemical analysis by Hikiba *et al.* show that the DMC1 M200V variant biochemically has reduced stability, impaired homology search and strand exchange activities, DNA-binding, and ATPase activity compared to wild type recombinant protein (53). Importantly, He *et al.* notes that the frequency of this *DMC1* variant in African and Latino populations is high (54). This variant on a pre-synaptic DMC1 structure (PDB 7C9C) highlights that the methionine is located between two alpha helices and the mutant valine likely affects alpha helical packing which modulates structure and function (Figure 1).

Analysis of *DMC1* gene status was performed by Wang *et al.* in a study of 192 Han Chinese women at the Hospital for Reproductive Medicine affiliated with Shandong University in China (55). Two SNPs were identified in *DMC1* introns: c.8632C > T in intron 4 and c.32377G > C in intron 10 (55). However, there were no significant differences found between POI patients and the control, indicating that there is likely no impact on protein function (55).

He *et al.* identified a third *DMC1* variant by performing whole exome sequencing of two infertile consanguineous siblings of a Han Chinese family at the in Reproductive and Genetic Hospital of CITIC-Xiangya in Changsha, Hunan, China (54). The male had non-obstructive azoospermia, and the female had POI with amenorrhea, low estradiol, high FSH, and ovaries with only one small follicle (54). Their analysis revealed a novel *DMC1* variant c.106G > A (D36N) that is likely pathogenic and a source of infertility in these patients (54). This mutation is predicted to affect DMC1 filament oligomerization (54). This is supported by a pre-synaptic DMC1 structure (PDB 7C9C) in which we highlighted in red the D36N mutation which is present within the N-terminal lobe domain of a DMC1 monomer (Figure 1).

In another study by Cao *et al.*, researchers examined mutational status of two daughters with diminished ovarian reserves but not POI and a son with non-obstructive azoospermia (56). This study was performed at the Reproductive Medicine Center at the University of Hong Kong-Shenzhen Hospital in Shenzhen, China to determine what genetic variants could play a role in the impaired fertility of this family (56). Cao *et al.* discovered a predicted pathogenic homozygous mutation in *DMC1* c.28delG (E10N) in these patients, resulting in a frameshift mutation in the DMC1 protein (56). Both daughters had reduced follicle counts, but without a diagnosis of POI, the study cannot determine if this specific DMC1 variant plays a role in POI pathogenesis (56). However, it is interesting to note that the fertility defects corresponding with this mutation were present in all affected family members. Unfortunately, this amino acid lacks electron density in structures solved to date.

**Figure 2.**
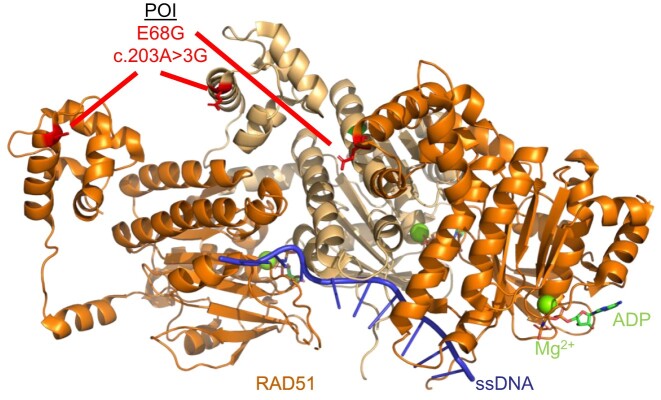
Schematic of the RAD51 (PDB 5H1B) pre-synaptic complex on ssDNA which shows the RAD51 protein protomer on ssDNA (orange and light orange). The RAD51B variant c.203A > 3G variant (E68G) is highlighted in red. ssDNA is depicted in blue, magnesium ions are shown as spheres in green, and ATP molecules are shown as sticks according to ion color with a green backbone.

**RAD51:** RAD51 is an essential gene that mediates high-fidelity repair during HR and replication stress (57–60). Luo *et al.* identified a *RAD51* variant c.203A > 3G (E68G) through whole exome sequencing of 50 POI patients who presented with primary amenorrhea, dysregulated hormone levels, and a lack of ovarian follicles from the Reproductive Hospital Affiliated with Shandong University in Jinan, China (25). This mutation results in a change in the RAD51 amino acid E68G which displayed cytoplasmic localization in HEK293T cells (25). Impaired nuclear localization of RAD51 likely results in a decrease of RAD51 functions associated with genome maintenance and oocyte development. Mapping of the E68G variant on a structure of RAD51 on ssDNA shows that this variant likely diminishes interaction with other RAD51 monomers (Figure 2).

**Figure 3.**
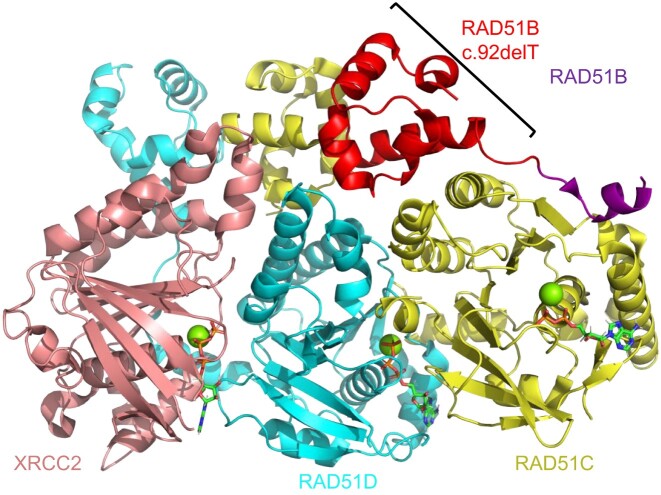
Schematic of the BCDX2 complex (PDB 8OUZ) which shows the RAD51B protein (purple), RAD51C protein (yellow), RAD51D protein (turquoise), and the XRCC2 (salmon) in cartoon. The RAD51B variant c.92delT which has 63 amino acids deleted from the N-terminus of the RAD51B protein is highlighted in red. Magnesium ions are shown as spheres in green and ATP molecules are shown as sticks according to ion color with a green backbone.

**RAD51B:** RAD51B is a RAD51 paralog in the BCDX2 complex that functions to stimulate RAD51 loading on ssDNA (10,11). The RAD51 paralog proteins are comprised of an N-terminal domain and an ATPase domain. The N-terminal domain is important for contacting the ATPase domain of adjacent RAD51 paralogs or RAD51 molecules. Interestingly, the N-termini of the RAD51 paralogs contain several methionine residues which allow multiple start sites and various isoforms. Searching the GWIPS-viz web server, which draws from published Ribo-seq profiles deposited in the UCSC Genome Browser, we identified multiple RAD51B start sites which are varied based on cancer cell type (61). Interestingly, an alternative RAD51B isoform has recently been identified in a POI by Franca *et al.*. Using whole genome sequencing, the authors identified a pathogenic homozygous variant in the *RAD51B* gene c.92delT in two Brazilian sisters with POI (31). Upon pelvic ultrasound at age 23 and 21, these sisters displayed a small uterus and lacked the presence of ovaries (31). Interestingly, this *RAD51B* variant c.92delT introduces a frameshift deletion within the protein and its alternative start site for protein expression occurs at M64. Deletion of the gene before amino acid 64 results in complete deletion of the N-terminal domain (NTD) of RAD51B. The N-terminal domain is important for anchoring RAD51B on RAD51C so that the dynamic C-terminal region of RAD51B can stimulate RAD51 loading as shown in PDB 8OUZ (Figure 3) (31). When re-capitulated in mouse models, the authors found defects at several stages of meiosis resulting in a reduction of crossover events (31). Additionally, the authors found that the *RAD51B* variant c.92delT in human-derived lymphoblastoid cells under fork stress conditions have diminished replication fork tracts and fork progression speed (31). Combined with the loss of its interaction with RAD51C and a partial loss of interaction with RAD51 and HELQ, this study provides definitive data showing that RAD51B is necessary in meiosis (31). These data highlight the importance of RAD51B function in female reproductive diseases and support previously published biochemical, biophysical, and structural studies indicating RAD51B is an dynamic paralog cruicial for stimulating RAD51 function (10). Combining the patient data, cellular characterization of this variant, and the biochemical and physical analysis indicates that RAD51B is a potential biomarker candidate for POI.

**SWS1 (ZSWIM7):** Zinc finger SWIM domain-containing protein 7 (SWS1, ZSWIM7) is a RAD51 regulator protein that is part of the functional SWSAP1-SWS1 heterodimeric complex and has been implicated in POI in three different studies (17,62,63). SWSAP1-SWS1 functions during replication restart via RAD51-dependent repair mechanism (17,62). The SWSAP1-SWS1 protein complex is a recombination mediator like BRCA2 and PDS5B that can stimulate RAD51-dependent recombination on RPA coated DNA (17).

McGlacken-Byrne *et al.* studied two sisters with POI at the University College London Hospitals (30). Both presented with symptoms consistent with POI including absent puberty, primary amenorrhea, elevated gonadotropin levels, low estradiol concentrations, and absent ovaries (30). The sisters underwent whole genome sequencing and a homozygous variant in *SWS1* c173C > G (S58*) was identified. This variant results in a premature termination codon (denoted as a star) at amino acid S58 which is predicted to undergo nonsense-mediated decay (64). *SWS1* gene expression was analyzed in fetal gonad development and higher expression was observed in ovaries compared to testes, suggesting an important role for *SWS1* in female germ cell meiosis (30). Additionally, qRT-PCR expression of *SWS1* in adult ovaries identified *SWS1* is present at all stages of reproductive development (30). While the role that the SWS1 protein plays in embryos and adult ovaries is unknown, it is likely tied to the stimulation of RAD51-dependent recombination (17).

Hussain *et al.* discovered another POI-related *SWS1* missense variant, c.176C > T (S59L), in a Pakistani family with both male and female reproductive complications (65). This *SWS1* variant c.176C > T (S59L) is similar in genomic position to the in *SWS1* variant found by McGlacken-Byrne *et al.* (30). The affected male members of this family presented with small testes and had non-obstructive azoospermia as well as elevated levels of LH and FSH (65). Females in this family were diagnosed with POI and presented with amenorrhea, smaller uteri, and ovaries without follicles (65). This mutation results in a SWS1 protein mutation at S59L which is close to the predicted zinc-finger domain. Importantly, this variant is absent in a database of ∼3000 Middle eastern alleles and their compared controls. The data in this study suggest that this *SWS1* variant is the cause of infertility and reproductive disease in this family. Given the proximity of the chromosomal mutations *SWS1* c.173C > G (65) and c. 175C > T (30) and their correlation to POI, these studies may have collectively identified a hotspot in human chromatin that is susceptible to mutagenesis.

A third independent study of three patients of Turkish ethnicity revealed a novel homozygous *SWS1* variant c.38T > C (L13P) in one family and two c.173C > G mutations in an unrelated family resulting in a stop codon at S58* (66). The patient with the SWS1 L13P variant presented with elevated FSH and LH levels, an absence of ovaries upon ultrasound, and a prepubertal uterus upon magnetic resonance imaging (66). The L13P mutation is in a predicted alpha helix, which severely distorts secondary structure predictions (66). There are currently no published structures of SWSAP1-SWS1.

**SPIDR (KIAA0146):** Another RAD51 modulator (SPIDR, KIAA0146) has been implicated in POI (28,62). SPIDR has been shown to interact with RAD51, SWSAP1-SWS1 and the BLM helicase, all important proteins involved in high-fidelity DNA repair (62,67). While this protein has been identified to be part of various DNA repair complexes, its own function has yet to be biochemically defined. Interestingly, three studies have identified premature stop codons in the *SPIDR* gene in patients diagnosed with POI from three different countries. The first study by Smirin-Yosef *et al.* identified the *SPIDR* variant c.839G > A in two sisters of Arab ancestry, both diagnosed with POI at the Pediatric Endocrine Clinic in Israel. Both sisters presented with a small uterus, one having an absence of ovaries while the other presented with one ovary without follicles (28). Exome sequencing revealed a SPIDR variant resulting in a premature stop codon at amino acid position W280* (28). Interestingly, cellular analysis of both patient mutations showed elevated levels of HR, single-strand annealing, and non-homologous end joining compared to wild type suggesting that SPIDR enables genome stability (28).

Using next-generation sequencing, Heddar *et al.* identified a novel homozygous *SPIDR* variant c.814C > T (R272*) in an Indian woman diagnosed with POI (68). The woman presented with primary amenorrhea, delayed puberty, and delayed bone development (68). Further characterization of the woman's lymphocyte cells identified an increase in DNA damage after mitomycin C (MMC) treatment compared to WT cells (68). Both of these premature stop codons studied by Smirin-Yosef *et al.* and Heddar *et al.* are in the N-terminus of SPIDR which is predicted to be unstructured on the IUPred2A server (69).

In a multi-center, nationwide study in France by Rouen *et al.*, 36 POI patients and their families were analyzed and identified another *SPIDR* variant at c.2002C > T resulting in a premature stop codon at amino acid position (Q668*) (70). It is unclear from these studies which isoform of SPIDR was sequenced but Q668 is in the C-terminal region of the protein which is predicted to be structured on the IUPred2A server (69). Given an absence of biochemical characterization of this protein, it will be interesting to see how these variants impact SPIDR function.

**XRCC2:** X-ray repair cross complementing 2 (XRCC2) is a RAD51 paralog in the BCDX2 complex (71). One *XRCC2* mutation at c.41T > C (L14P) was identified by Yang *et al.* in azoospermia and was later confirmed by the same group that this *XRCC2* variant can lead to the development of POI as well (72,73). The *XRCC2* variant c.41T > C (L14P) was identified in a Chinese family where infertile siblings underwent whole exome sequencing (72). At age 16, the sister presented with amenorrhea, a small uterus, small ovaries, high levels of gonadotropin hormones, and low E2 and AMH serum concentrations (72). The brother was diagnosed with non-obstructive azoospermia (72). The consequence of the *XRCC2* variant c.41T > C (L14P) is a result of aberrant splicing of the protein (72). Testing spermatocytes of the male patient using the terminal deoxynucleotidyl transferase dUTP nick end labeling (TUNEL) assay found an increase in apoptotic cells, suggesting a possible molecular consequence of the *XRCC2* variant in POI (72).

**BRCA1:** Breast cancer type 1 susceptibility protein (BRCA1) is an important protein that functions after resection in the initial steps of HR with PALB2 and BRCA2 to commit repair to HR. Although variants have yet to be identified in POI, BRCA1 mutational and functional status may be an indicator of ovarian reserve and menopause. Wang *et al.* explored the correlation between *BRCA1* germline mutations and functional oocytes. They found that *BRCA1* mutant carriers had statistically significant decreased AMH levels which are indicative of a decreased ovarian reserve and infertility and are diagnostic criteria of POI (74). Additionally, Turan and Oktay found that BRCA1 and RAD51 activity declined as oocytes matured after age 37 (33). Women with *BRCA1* mutations have increased DSBs in their oocytes which correlates with a loss of primary follicles and early onset of menopause (33,38).

**BRCA2:** Breast cancer type 2 susceptibility protein (BRCA2) is a tumor suppressor and recombination mediator which loads RAD51 on RPA-coated ssDNA. Both BRCA1 and BRCA2 proteins are commonly mutated in female reproductive cancers (3,18), so it is likely that there is a connection to their possible mutation in reproductive diseases. Due to its role in HR, BRCA2 activity is likely necessary during the crossover generation phases of meiosis due to an increase in recombination activity; it has been shown in human fetal ovaries that the highest expression of BRCA2 mRNA occurred during meoitic prophase I, specifically seen in the pachytene stage by immunostaining (33,75). Weinberg-Shukron *et al.* studied the *BRCA2* status of two Ethiopian sisters with POI (75). The sisters symptoms included primary amenorrhea, absence of puberty, no detectible uterus or ovaries and abnormal gonadotropin levels (75). Following oral estradiol and then estrogen-progesterone treatment, both sisters had secondary sexual characteristics, adult-size uteri and regular menstrual periods (75). With whole exome sequencing of the sisters and members of their family, Weinberg-Shukron *et al.* discovered that the sisters had compounding *BRCA2* variants which presented heterozygously: the *BRCA2* variant c.7579delG (V2527X) and the *BRCA2* variant c.9693delA (S3231fs16*) (75). Transcript and protein analysis of these variants showed reduced levels compared to an unrelated and healthy control (75). The V2527X variant was within the BRCA2 helical domain (DNA binding domain) and the S3231fs16* variant follows the C-terminal NLS is predicted to truncate the remaining 171 amino acid residues. Their mother was the carrier of *BRCA2* c.7579delG variant and was eventually diagnosed with ovarian cancer, further indicating a role of this specific *BRCA2* variant in ovarian disease pathogenesis (75).

**Figure 4.**
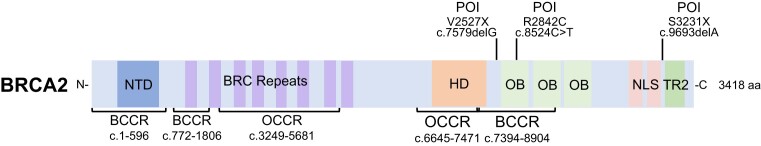
Linear schematic of the BRCA2 protein highlighting breast and ovarian cancer cluster regions and mutations associated with reproductive disorders. The BRCA2 protein is shown as a 3418 amino acid protein with an N-terminal DNA binding domain (NTD), eight BRC repeats, a helical domain (HD), three oligonucleotide-binding folds (OB), two nuclear localization sequences (NLS) domains, and a RAD51 interacting site (TR2). BRCA2 has three BCCRs from c.1–596, c.772–1806, and c.7394–8904 and one OCCR from c.1380–4062. The POI *BRCA2* variants c.7579delG and c.8524C > T are in BCCRs and c.9693delA (S3231X) variant within the C-terminus of the BRCA2 protein.

In a study by Caburet *et al.*, another *BRCA2* variant was found exclusively in a POI patient not associated with Fanconi anemia or cancer (76). Exome sequencing of this patient and her family concluded that she had a *BRCA2* missense variant c.8524C > T (R2842C) (76). This *BRCA2* variant is predicted to be pathogenic and alters an amino acid in the ssDNA-binding of the tower domain of BRCA2 (76). Caburet *et al.* further investigated the effect of the *BRCA2* variant c.8524C > T on HR. After silencing endogenous *BRCA2* in human RG37 cells, they found that expression of the mutated BRCA2 protein only partially rescued HR in the cells back to normal wild type BRCA2 levels (76). All variants are mapped on the BRCA2 protein schematic in Figure 4. It is interesting to note that two of the three variants (V2527X and R2842C) are located within one of the breast cancer cluster regions (BCCR) in BRCA2 (77).

**Endometriosis:** Endometriosis is a disease that is estimated to affect about 10% of reproductive-age women worldwide, which equates to ∼190 million people (78). In the United States specifically, ∼6.5 million women are currently living with endometriosis and 1 in 10 women are affected by the disease (79–81). Endometriosis is characterized by excessive growth of endometrial tissue outside of the uterine cavity, causing scarring and lesions that can affect menstruation, sexual intercourse, and fertility (82). Aside from the reproductive effects of this disease, other symptoms can include fatigue, mood disorders, cardiovascular diseases, gastrointestinal and urological problems. Additionally, this disease can affect personal, social, educational, and occupational contexts that severely diminish an individual's quality of life. Endometriosis can also lead to the development of breast and ovarian cancer (79,82).

The State of Current Endometriosis Research: Current research fails to define the causative factors of endometriosis and there are no screening tools or definitive biomarkers that are validated for the identification of this disease. Genetic databases that identify mutations related to human diseases overwhelmingly lack data from endometriosis patients (83). Without combined characterization of and access to endometriosis-associated genomic, proteomic, and metabolomic profiles, research in this area is limited. Importantly, we do know that endometriosis predisposes patients to clear cell carcinoma and endometrioid ovarian carcinoma (20,21,84–87). This implies that endometriosis patients may have similar genetic defects, although we are still limited in our basic knowledge of endometriosis compared to other diseases.

Current Diagnostic and Treatment Practices for Endometriosis: There are many challenges in the current diagnostic practices for endometriosis. Many reproductive disorders have similar presentations and symptoms, often leading to misdiagnosis with an average of ∼10 years until diagnosis (79). Further difficulty in diagnosis is due to the fact that endometrial tissue must be observed in other areas of the body outside of the uterus. This often requires invasive imaging and surgery, which present risks during and after operation (88). This may cause those with more mild cases to opt out of diagnostic confirmation which ultimately results in an underrepresentation in the population data of this disease.

Although endometriosis is diagnosed when endometrial tissue grows outside of the uterine cavity, there are a variety of symptoms that affect patient quality of life, seemingly separate from the disorder's gynecological origin. These include cardiovascular and autoimmune complications, mood and energy fluctuations, as well as gastrointestinal and urological problems (82). Finding one treatment strategy that will treat all endometriosis symptoms is difficult. Typically, patients undergo surgery followed by a combinatorial treatment including hormonal contraceptives and gonadotropin-releasing hormone (GnRH) for management (88). In extreme cases, a hysterectomy can be performed (88). Treatment for endometriosis is also complicated by the high rate of recurrence of endometrial lesions (88). It has been reported that within 5 years of surgery, there is a 50% chance of patients requiring additional surgery for recurring lesions outside of the uterus (88). As this disease is persistent across the patient's lifetime, surgical treatments are often repeatedly required.

Endometriosis and DNA Repair: The main barriers to endometriosis diagnosis and treatment include a lack of biomarker and target identification, as the molecular etiology of the disorder is not fully understood. Some genetic mutations and variations in protein expression in various DNA repair pathways have been identified in endometriosis patients, indicating that there is likely a molecular basis to its etiology (21,84–87).

**XRCC3:** XRCC3 is a RAD51 paralog that comprises part of the CX3 complex (12). XRCC3 promotes HR through direct interaction with RAD51 as well as functioning in the CX3 complex (18,89). Attar *et al.* isolated genomic DNA from peripheral blood of 52 women with endometriosis and 101 women who did not have endometriosis to determine SNPs via PCR (90,91). The authors identified XRCC3 T241 polymorphisms that resulted in either T/T, T/M,T/M + T/T or T/M + M/M at the 241 position (90) as shown in (Table 2). Compared to controls, T/T was the only variant significantly increased with a (*p* = 0.018) (90). Interestingly, this study postulates that T/M heterozygous genotype (*p* = 0.022) may have protective roles against endometriosis via an unknown mechanism (90). Results from Attar *et al.* provide a novel link between endometriosis and polymorphisms in *XRCC3* which warrants further study.

**Table 2.** Mutations in RAD51 regulators in endometriosis

Gene symbol	Protein	Alteration in disease	Reference
*XRCC3*	XRCC3 (X-ray repair cross complementing 3)	T/T genotype at amino acid position 241	Attar *et al.* (90)
*BRCA1*	BRCA1 (Breast cancer type 1 susceptibility protein)	c.3232T > A	Goumenou *et al.* (85)
		rs71361504 polymorphism (-/GTT)	Govatati *et al.* (86)
*CSB (ERCC6)*	CSB (Cockayne syndrome group B, Excision repair cross-complementation group 6)	rs2228528 polymorphism (GG > AA)	Shen *et al.* (95)

This table contains information about genetic variants implicated in endometriosis including gene and protein name, alteration in disease, and the references for each study.

**Figure 5.**
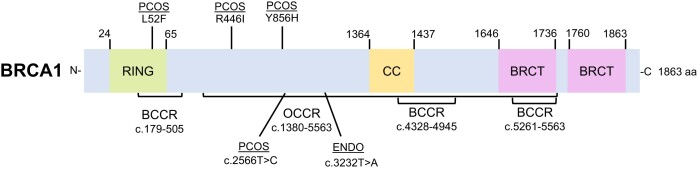
Linear schematic of the BRCA1 protein highlighting breast and ovarian cancer cluster regions and variants associated with reproductive disorders. BRCA1 is an 1863 amino acid protein with an N-terminal Really Interesting New Gene (RING) domain for DNA binding, a coiled-coil (CC) domain, and two BRCA1 C-terminal (BRCT) domains. BRCA1 has three breast cancer cluster regions (BCCR) from c.179–505, c.4328–4945 and c.5261–5563 and one ovarian cancer cluster region (OCCR) from c.1380–4062. The *BRCA1* variants c.3232T > A found in endometriosis (ENDO) and c.2566T > C found in PCOS are both located within the BRCA1 OCCR.

**BRCA1:** Goumenou *et al.* studied a family in Greece comprised of several members diagnosed with endometriosis, many of whom underwent surgical hysterectomy (85). Only one of the family members had an identified single nucleotide substitution in *BRCA1* at c.3232T > A of the exon 11 region as shown in (Table 2) (85). Daughters in this family consequently had dysmenorrhea and infertility (85). Nunes *et al.* describes regions of the BRCA proteins where mutations correlate with an increased risk of breast or ovarian cancer, known as the breast cancer cluster regions (BCCR) or ovarian cancer cluster regions (OCCR) (77). Interestingly, this *BRCA1* c.3232T > A variant found associated with endometriosis is located within an OCCR (c.1380–4062) of *BRCA1* and mutations in this region of *BRCA1* are known to be characterized as pathogenic in ovarian cancers (Figure 5) (85).

Govatati *et al.* examined how mutations in *BRCA1* impacted endometriosis in a study of 573 Indian women with the disease from two ethnically different linguistic groups from the Infertility Institute and Research Centre (IIRC) in Secunderabad, India and the Institute of Reproductive Medicine in Kolkata, India. The authors found that the *BRCA1* polymorphism rs71361504 (-/GTT) was significantly associated with the risk of endometriosis (*p* value < 0.0001) as highlighted in (Table 2) (86). Further characterization of this *BRCA1* variant in eutopic endometrial tissue of endometriosis patients and controls identified significantly decreased BRCA1 protein expression as well as a loss of nuclear localization of the protein in patients with endometriosis (86).

**CSB (ERCC6):** Cockayne syndrome Group B (CSB, also known as ERCC6) has been canonically described to function in nucleotide excision repair (NER) and transcription coupled-nucleotide excision repair (TC-NER). Recently, CSB has been identified to moonlight with HR proteins to repair R-loops via RAD51-dependent and RAD52-dependent mechanisms (92–94). Shen*et al.* discovered a polymorphism in *CSB* that was associated with endometriosis. The authors studied *CSB* mutations in 153 endometriosis patients at the Chung Shan Medical University Hospital in Taiwan, most of whom had advanced stages endometriosis (95). They found that the *CSB* rs2228528 polymorphism (*p* = 0.0055) resulting in a GG > AA genotype alteration associated with a higher risk of endometriosis as shown in (Table 2) (95). Further mechanistic studies to understand the effect of this mutation will be important for understanding its role in endometriosis and its contributions in repair.

**Polycystic ovary syndrome:** Polycystic ovary syndrome (PCOS) is a reproductive endocrine disorder that is described by enlarged polycystic ovaries, high levels of androgenic hormones, irregular menstruation, dysregulation of ovulation, and insulin resistance (96,97). Globally, PCOS affects ∼4–21% of reproductive-age women depending on diagnostic criteria (98–101). The main symptoms of PCOS include hyperinsulinemia, anovulation or oligoovulation, amenorrhea, dysmenorrhea, hyperandrogenemia, secondary hirsutism, and the presence of cystic ovaries (96). The etiology of this disease is unknown. However, it has been proposed that it can be caused by genetic and epigenetic mutations or modifications, exposure to environmental toxicants, chronic inflammation, and/or neuroendocrine dysfunction (96,102). Understanding the etiology of this disease is important as PCOS is a risk factor for cardiovascular diseases, type II diabetes, endometrial cancer, ovarian cancer, and pancreatic cancer (103–107).

Current Diagnostic and Treatment Practices for PCOS: Current diagnostic practices for PCOS have been established together by the European Society of Human Reproduction and Embryology (ESHRE) and American Society for Reproductive Medicine (ASRM) and are referred to as the Rotterdam criteria (108). The Rotterdam criteria is the most widely accepted diagnostic tool for PCOS (109,110). The criteria diagnose PCOS when a patient presents with two out of the three major symptoms of the disease: hyperandrogenemia, ovulatory dysfunction, or polycystic ovaries (98,109–111). Typical presentations include high levels of luteinizing hormone (LH), insulin, androgen, and AMH and lower FSH levels in PCOS (96).

The most common treatments for PCOS are categorized into three main groups: hormonal, metabolic, and lifestyle modifications. A varied combination of all three treatment strategies is used over a patient's lifetime. Patients are often treated hormonally with oral contraceptives to regulate menstrual and ovulatory cycles as well as to treat secondary hirsutism or excessive facial hair growth (111). To treat metabolic symptoms, metformin is often prescribed to manage insulin resistance and hyperandrogenism as well as protect against the development of cardiovascular diseases (103,111,112). In addition, lifestyle factors are also used to manage the hormonal and metabolic symptoms. These lifestyle factors include diet and exercise modifications (96,111,113,114).

The State of Current PCOS Research: The molecular mechanisms underlying PCOS are poorly understood. There are currently no biomarkers used for disease prognosis before puberty (115). A variety of genes are thought to play a role in the development of PCOS, however, it is likely that a combination of genetic, epigenetic, and developmental toxicant exposures has equal importance in disease etiology, complicating molecular research (115). Unfortunately, there are no cures for PCOS, and treatment strategies fall short in their effectiveness to manage a patient's diverse symptoms. It is necessary to develop a deeper understanding of the molecular pathogenesis of this disorder to improve treatment options.

**DNA damage and repair in PCOS:** PCOS is a highly heritable disorder and there have been several loci that are associated with the disease, some discovered in DNA repair genes (96,116). We highlight two polymorphisms and three missense mutations in *BRCA1* as shown in (Table 3).

**Table 3.** Mutations in RAD51 regulators in polycystic ovarian syndrome PCOS

Gene symbol	Protein	Chromosomal mutation or polymorphism	Protein mutation	Mutation effect	Reference
*BRCA1*	BRCA1 (Breast cancer susceptibility protein type 1)	rs71361504 -/GTT	N/A	N/A	Siddamalla *et al.* (118)
		rs3092986 T/C	N/A	N/A	
		c.154C > T	L52F	missense	Jiao *et al.* (119)
		c.1337G > T	R446I	missense	
		c.2566T > C	Y856H	missense	

This table contains information about variants implicated in PCOS including gene and protein name, chromosomal location, protein mutation (if known), functional effect of the protein variant (if known), and the references for each study.

**BRCA1:** BRCA1 has been described to have effects on metabolism, steroidogenesis, and the activity of steroid hormones including estrogens and androgens (117). It is likely that BRCA1 function can have an impact on PCOS development due to the highly metabolic nature of this disease. In a study of 110 South Indian women diagnosed with PCOS, Siddamalla *et al.* identified the presence of two common *BRCA1* SNPs, rs71361504 -/GTT and rs3092986 T/C (118). Both polymorphisms are in the *BRCA1* promoter.

One of the characteristics of PCOS is that patients often experience longer, more irregular menstrual periods (119). Jiao *et al.* studied 20 patients with PCOS, 10 of which had clinically normal periods and 10 with irregular periods (119). They investigated how profiles such as DNA methylation, sequencing and expression compared between groups using whole genome sequencing (119). Their sequencing revealed that there are three *BRCA1* variants associated with PCOS in the irregularly menstruating patients: c.154C > T (L52F), c.1337G > T (R446I) and c.2566T > C (Y856H) (119). It is mentioned that the *BRCA1* variant c.154C > T (L52F) is located within an N-terminal interaction region of BRCA1 and is inactive for ubiquitin ligase activity (119–124). Interestingly, studies in mammary epithelial cells revealed that BRCA1 L52F was wild type for HR function but was only able to pair centrioles and was defective in centrosome duplication (125). Recently, CRISPR-mediated base editing methods (BE3) in HAP1 cells determined that the BRCA1 L52F mutant is indeed a pathogenic mutation as it is defective in homology directed repair (126). It will be important to determine if these same mechanisms are maintained in patient derived cells with PCOS.

## Discussion

The molecular origins and mechanisms of female reproductive disorders in the context of DNA repair are still unknown. Specifically, how the RAD51 regulators intersect reproductive disease etiology is under-studied. Of the three reproductive disorders we examined, POI has the largest dataset on RAD51 modulators, while endometriosis and PCOS are severely lacking. Even with the limited data in the literature, we can conclude that researching mutations in the RAD51 regulators has potential to progress our molecular understanding and therapeutic innovation for patients, as many of these mutants have severe phenotypes. In addition, identifying variants in regulators of DMC1 such as HOP2 and MND1 will be essential for understanding meiotic regulation in these diseases. Advancement in this area is important for many reasons including time to diagnosis for patients often takes years and treatments have undesirable side effects.

Additionally, there are inadequacies in screening tools and biomarkers that inhibit advancement of our understanding of these diseases. Given that the wild type function of many of these DNA repair proteins are currently being characterized, it is not surprising that the genes we suggest using as biomarkers include few genes that have extensively been characterized *in vitro* and *in vivo*. For example, given the combined cellular and functional inhibition of the L52F variant in BRCA1 identified in PCOS, we suggest this should be a novel biomarker for this disorder. It remains unknown if the RAD51 paralog mutants identified in RAD51B, XRCC2, XRCC3 and SWS1 (required for stability of the RAD51-paralog containing complex with SWSAP1) which could remain proficient for HR, may have a separate importance in these female reproductive diseases through inhibition of other protein activities. While there lacks extensive biochemical and cellular data on SPIDR, given that it was identified in three independent studies of POI patients from different continents, suggests a probable impact on POI development and could be used as a genetic biomarker of this disease. Knowing the recent important function of SWS1-SWSAP1 in template switching and playing a recombination mediator role, we also suggest that SWS1 be included as a genetic biomarker for POI.

It is important to note that three mutations in *BRCA1* or *BRCA2* from our analysis of reproductive disorders are found within known pathogenic mutational regions of these genes (Figures 4 and 5). The *BRCA1* variants in endometriosis (c.3232T > A) and PCOS (c.2566T > C) were identified within an OCCR of the gene and two *BRCA2* variants in POI (c.7579delG, c.8524C > T) were identified to be within BCCR regions of the gene (77). We believe that the mutants’ association with the cancer cluster regions could provide clues to disease severity, progression, or predisposition to breast or ovarian cancer.

Another interesting topic involves fertility (AMH levels) and *BRCA* status. Dreschel *et al.* explored the relationship between ovarian reserve using AMH levels and follicle counts with *BRCA* status in 36 *BRCA* mutational carriers and 126 controls over the course of a 5-year multinational study (127). They did not find any significant differences in ovarian reserves between the mutated and control study groups (127). Another study by Lin *et al.* examined germline *BRCA* mutations and ovarian reserve using ovarian tissue from *BRCA* mutation carriers and donated cadaver ovarian tissue (128). There were 13 patients who were *BRCA1*mutation carriers, 5 patients who were *BRCA2* mutation carriers and 12 organ donor cadavers with healthy ovaries were used as controls; all subjects were free of malignancies (128). *BRCA* mutation carriers showed a significant decrease in follicle density and a significant increase in DNA damage within the follicles using yH2AX staining compared to the controls (128). Interestingly, DNA damage in the ovarian follicles of the *BRCA* mutation carriers increased exponentially with age (128). Overall, this study shows that *BRCA* status may be an indicator of ovarian health, aging and fertility. These studies however do not mention specific *BRCA* mutations and often contradict each other highlighting the complexity of studying fertility and reproductive diseases.

Another consideration when examining the role of DNA repair genes and reproductive diseases is the connection to reproductive senescence. Once oocyte pool depletion occurs, it increases the risk of uterine and ovarian cancer by 42% in women aged 55 and older (129–131). Interestingly, variant analysis of menopausal women identified two-thirds of the variants occur in DNA damage response genes (132,133). A new bioRxiv preprint by Osei B. et al. (2024) describe a heterozygous HELB variant (rs75770066). HELB is a helicase that unwinds DNA in a 5′ -to-3′ polarity, removes RPA from ssDNA, promotes entry into S phase of the cell cycle, and increases cell survival from replication stress (134–138). The HELB (D506G) variant displays decreased RPA interaction, reduced DSB recruitment, and patients with a homozygous variant have decreased ovarian reserve and an earlier onset of menopause. It is clear from this work that understanding the connection between DNA damage response proteins and the link to reproductive senescence is an important new emerging field.

Overall, the RAD51 regulators are not only important for DNA repair and genetic stability in all cells but are specifically involved in processes occurring in the earliest stages of the developing female reproductive system. Mutations in the RAD51 modulators are particularly well-documented in reproductive cancers and therefore they likely play a direct role in the development of reproductive disorders like POI, endometriosis, and PCOS. This area of research is an opportunity to address an unmet need for personalized, targeted therapies for patients, provide potential novel biomarkers, decrease cost of healthcare, and improve the quality of life of those with reproductive disorders.

## Data Availability

The data underlying this article will be shared on reasonable request to the corresponding author

## Notes

Present address: Sarah R. Hengel, Department of Biology, Tufts University, Medford, MA 02155, USA.
